# Dogs (*Canis familiaris*) Gaze at Our Hands: A Preliminary Eye-Tracker Experiment on Selective Attention in Dogs

**DOI:** 10.3390/ani10050755

**Published:** 2020-04-26

**Authors:** Tadatoshi Ogura, Mizuki Maki, Saki Nagata, Sanae Nakamura

**Affiliations:** School of Veterinary Medicine, Kitasato University, CityTowada, Aomori 034-8628, Japan

**Keywords:** attention, dog, eye tracking, non-verbal communication, visual attention

## Abstract

**Simple Summary:**

Dogs seem to communicate with humans successfully by getting social information from body signals such as hand signs. However, less is known regarding how dogs pay attention visually toward human body signals including hand signs. The objective of this pilot study was to reveal dogs’ social visual attention tuned to inter-species communication with humans by comparing gazing patterns to the whole body of human, dogs, and cats. The pictures showing humans with or without hand signs, dogs, and cats were presented on a liquid crystal display monitor, and gazing behaviors of subject dogs to these pictures were recorded by an eye-tracking device. The subjects gazed at human limns more frequently than limbs within conspecifics and cat images, where the dogs attention were focused on the head and body. Furthermore, gaze toward hands was greater in the human hand sign photos relative to photos where human hand signs were not present. These results indicate that dogs have an attentional style specialized for human non-verbal communication, with an emphasis placed on human hand gestures.

**Abstract:**

Dogs have developed a social competence tuned to communicate with human and acquire social information from body signals as well as facial expressions. However, less is known regarding how dogs shift attention toward human body signals, specifically hand signs. Comparison among visual attentional patterns of dogs toward whole body of human being, conspecifics, and other species will reveal dogs’ basic social competences and those specialized to inter-species communication with humans. The present study investigated dogs’ gazing behaviors in three conditions: viewing humans with or without hand signs, viewing conspecifics, and viewing cats. Digital color photographs were presented on a liquid crystal display monitor, and subject dogs viewed the images while their eyes were tracked. Results revealed that subjects gazed at human limbs more than limbs within conspecific and cat images, where attention was predominately focused on the head and body. Furthermore, gaze toward hands was greater in the human hand sign photos relative to photos where human hand signs were not present. These results indicate that dogs have an attentional style specialized for human non-verbal communication, with an emphasis placed on human hand gestures.

## 1. Introduction

Dogs’ visual cognition has long been a popular research topic. During their long history of domestication, dogs have developed a human-social skill (for a review see [1]). For example, in an assessment of visual social cognition, Pitteri et al. [2] described that dogs can discriminate between an owner and strangers based on isolated internal features of a human face (i.e., eyes, nose, or mouth), with configural information being highly important. During the process of domestication, dogs have also been “worked” under various situation (for a review see [3]), so that they acquired the ability to discern social information from human body signals. Dogs successfully follow human pointing gestures at a much younger age than their closest wild-living relative (i.e., wolf, *Canis lupus*), even when hand-reared [4]. Furthermore, dog-owners use a variety of hand signs as non-verbal signals toward their dogs, which provide cues for following human intentions; however, little is known as to dogs’ attentional patterns to these hand signs.

Visual attention across various animal species has recently been investigated through eye-tracking techniques. Eye-trackers are able to record subtle gaze movements, enabling researchers to detect small changes in subjects’ interest and/or focal attention (for a review see [5]). Additionally, task-specific pre-training is not required; therefore, naturalistic behavior toward stimuli can be investigated. For example, one eye-tracking study revealed that among chimpanzees (*Pan troglodytes*), facial features are prominently detected when viewing pictures of both chimpanzees and humans [6]. Specifically, when exposed to photographs of conspecific and human faces, chimpanzees intensely focus on eye and mouth regions [7]. Gorillas (*Gorilla gorilla*) and orangutans (*Pongo abelii*) show similar attentional biases to facial images [8]. For non-primate animals, Schwarz et al. [9] developed a head-mounted eye-tracking system for chickens (*Gallus gallus domesticus*), while Tyrrell et al. [10] developed a system for starlings (*Sturnus vulgaris*) and was successfully able to detect valid eye movements. A head-mounted eye-tracker was also used in a group of peahens (*Pavo cristatus*), revealing gaze preferences toward the lower train relative to the head, crest, and upper train of peacocks [11]. Eye-trackers have also been utilized with dogs, especially for purposes of investigating human face processing. For instance, Somppi et al. [12] successfully employed an eye-tracker to investigate visual cognition in a sample of privately owned dogs. The authors found that dogs preferred facial images of conspecifics relative to human faces, children’s toys, and alphabetic characters, while also fixating longer on a familiar image relative to novel stimuli. In a related study, kennel and privately owned dogs showed greater interest in eye relative to adjacent face regions when viewing either conspecific or human faces [13]. Their interest in eye region were not found in inverted faces, suggesting that dogs are able to recognize both conspecific and human faces in photographs. More recently, Somppi et al. [14] observed that kennel and pet dogs gaze toward conspecific and human faces differently depending on the emotional expression displayed. Furthermore, oxytocin treatment can change pet dogs’ preferential looking patterns toward human facial emotional images, particularly within the eye region [15]. Téglás et al. [16] also revealed that pet dogs’ gaze follows a human’s referential signals (head-turning). Privately owned and kennel dogs gaze longer at human actors within social settings as compared to when viewing other dogs, which could indicate that processing social interactions within a non-conspecific context may be more demanding for dogs [17]. Although these frontier studies have expanded knowledge as to visual attention among dogs, research on gaze toward bodily signals outside a human face has not been conducted.

Human beings have been the most influential social partners for domestic dogs during their domestication process. Comparison among visual attentional patterns of dogs towards human beings, conspecifics, and other species will reveal dogs’ basic social competences and those specialized to inter-species communication with humans. The present study investigated dogs’ visual selective attention to human bodily signals, conspecifics, and cats (a species which lives in the same community with dogs but seems to be less influential to dogs’ attentional patterns than humans). If the dogs’ human-social skills have been tuned to recognition of human bodily signals, their gaze to human hands are more evident than the gaze to limbs of other species. We also examined the effect of stimulus familiarity when dogs viewed pictures of hand signs expressed by humans. The goal of this study was to determine how dogs’ fixation patterns could provide insight into cross-species communication with humans.

## 2. Materials and Methods 

### 2.1. Subjects

The subjects were 7 privately owned dogs, aged one to 18 years (Table 1). Before participating in this study, all dogs were trained by their owner to lie on a floor until commanded to move. Prior to the experimental protocol, the dogs visited the experimental rooms over at least two separate days and were trained to settle calmly, without the aid of their owner, and to pass a calibration test (see below) conducted by three experimenters (two handlers and one operator). During training, the dogs were not encouraged to fix their eyes toward any monitor or images. All training procedures were conducted based on shaping methods with positive reinforce training [18]. Four subjects did not pass the calibration procedure; thus, only data from 3 dogs were analyzed.

### 2.2. Apparatus and Experimental Set ups

The apparatus included an eye-tracking system (model ETL-300-HD, ISCAN, USA), which consisted of two computers, two color displays, and an eye-tracking camera. One computer and display was used to run the eye-tracking system and record eye movement data. The computer and display (43.2 × 32.4 cm, 1600 × 1200 px; model S2133-HBK, Eizo Nanao, Ishikawa, Japan) were used for stimulus presentation. The camera obtained images of each subject’s head and eyes. The camera emitted infrared light in order to track subjects’ monocular pupil and corneal reflection at 60 Hz.

**Figure 1:**
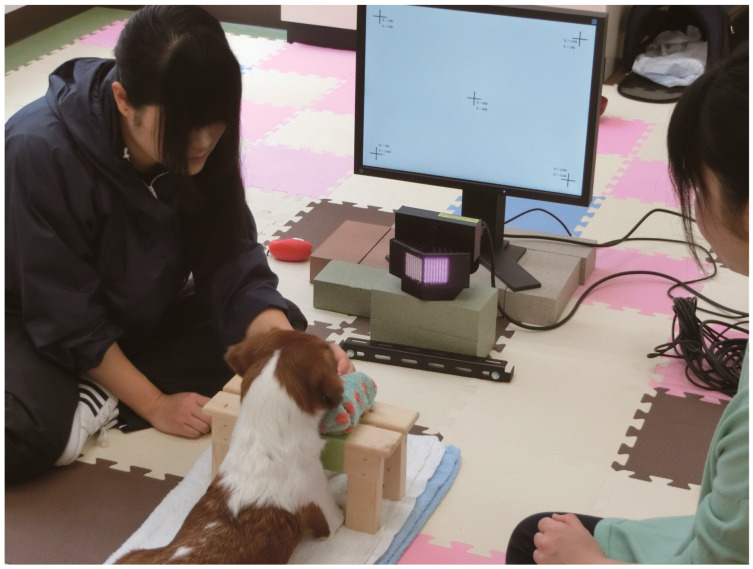
Subject dogs lied down in front of an eye-tracking camera and display. During calibration and the experimental session, the subject put his/her jaws on a purpose-designed chin rest.

All assessments were conducted in an experimental room at the School of Veterinary Medicine, Kitasato University, by one operator and two handlers (Figure 1). During the experiment, the dogs were released from a leash and could move around the room freely. The handlers guided the dogs to lie down on the floor in front of the presentation display; once the dogs complied, the dogs were rewarded with a treat. The distance between each dog’s head and the display was approximately 50 cm. During the calibration and experimental presentation, each dog was positioned between the two handlers staying closely with the dog. One of the handlers instructed each dog to put his/her jaw on a chinrest. The other handler lightly held the dog’s body if needed. The operator in the same room controlled the two computers. During the experimental procedure, the owners were absent from the room.

### 2.3. Stimuli

**Figure 2:**
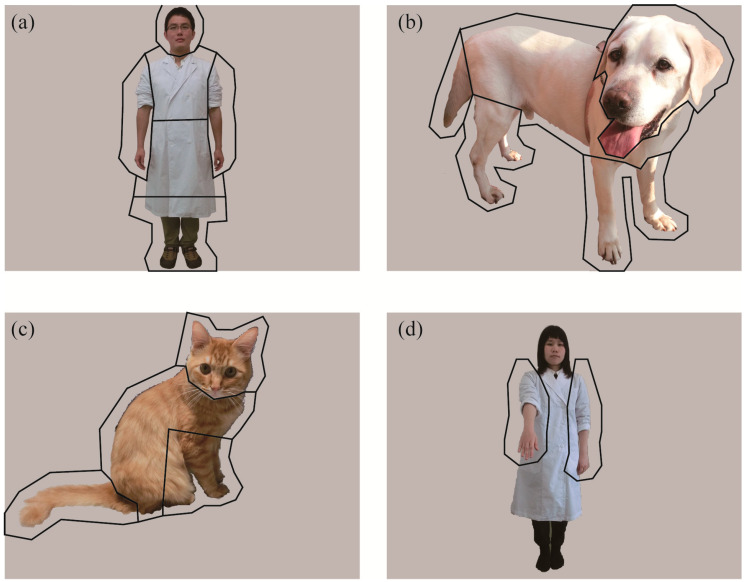
Examples of stimuli used in the experiment and areas of interest (AOI) employed in the analyses: (**a**) human without a hand-sign, (**b**) dog, (**c**) cat, and (**d**) human with a hand-sign. The frames drawn on the pictures (**a**–**c**) indicate the AOI utilized in the analysis comparing the three species and on the picture (**d**) indicates the AOI utilized in the analysis comparing stimuli with and without a hand-sign.

Digital color photographs comprising three stimulus species—humans, dogs, and cats—were used in the experiment (Figure 2). For each species, 12 images per subject were presented. All pictures were 1600 × 1200 px and consisted of single, whole body images of an individual on a gray background. The human pictures consisted of 3 novel individuals with a hand sign, 3 novel individuals without a hand sign, 3 familiar individuals with a hand sign, and 3 familiar individuals without a hand sign. A familiar person was defined as someone who spent time with the subject dog for longer than one day, including the owners, operator, and handlers. Hand signs used commonly in the subjects (“hand”, “sit”, or “stay”) were depicted. All human pictures were front facing, upright, and laterally symmetric except a hand showing a sign to avoid visuospatial attentional bias [19]. Each human model wore a white, laboratory coat for consistency and to avoid color distraction. The human sex was counterbalanced. All dog/cat pictures were novel to the subject dogs and were taken from a front/lateral side with a natural posture. Dog and cat breeds varied across the images. We constructed 6 sets of stimuli, comprising 6 pictures each, with at least one picture from each stimulus species.

### 2.4. Experimental Procedures

First, a 5-point calibration was conducted for each subject. Five black crosses were presented at the center, upper left, upper right, bottom left, and bottom right of the monitor on a gray background. A handler pointed to the crosses in that order using a treat. When the handler confirmed that the subject gazed on the cross, a vocal cue was provided to the operator in order to record the calibration point. This was repeated for the remaining 4 crosses. The calibration was then checked with a validation procedure: the handler pointed to the crosses again and the operator confirmed that the gaze points were on the crosses through the eye-tracker system. The calibration was repeated until all gaze points were validly recorded. 

After the calibration, the experimental session began. Each subject performed 6 sessions of the task. Each session began with a blank gray screen. When the subject gazed anywhere on the screen (not limited to any specific point to avoid the bias on the first fixation point described below), one of the handlers provided a vocal cue for the operator to start the stimulus presentation. A picture was then presented for 1.5 s, followed by a blank screen for 0.5 s. This routine was repeated for six times and progressed automatically by a computer program assembled for this experiment. Image presentation order was randomized. After each session was completed, the subject was rewarded with a treat, regardless as to whether the dog gazed at the screen. Each subject was then allowed to move freely around the room after each session to avoid to be stressed or habituated. Before the next session started, the subject was guided to lie down at the same position and the calibration was conducted again.

This work complied with the laws of Japan, and the experiment was approved by the President and Institutional Animal Care and Use Committee of Kitasato University (Approval no. 15-067).

### 2.5. Data Analyses

**Figure 3:**
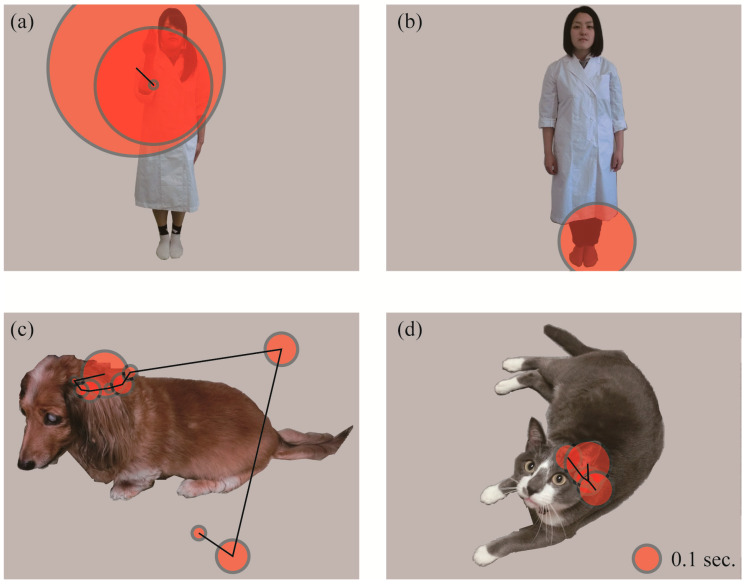
Examples of subjects’ gazing patterns: (**a**) human with a hand-sign, (**b**) human without a hand-sign, (**c**) dog, and (**d**) cat. Typical fixations and saccades are drawn on the pictures. The circles indicate fixations. The larger the circle, the longer the subject gazed in that area. The lines represent a saccadic path.

We divided each picture into several features (areas of interest; AOI) to quantitatively analyze subjects’ gaze patterns. For the analysis comparing the three species and that comparing human familiarity, the human/dog/cat within each picture was divided into four AOIs: Head, body, buttocks, and limbs (Figure 2a–c). For human pictures, the body indicated the upper third area from the neck to the feet, because the dividing bounds between body parts were obscure due to a white lab coat. The middle third area was included in the buttocks, and the bottom third and arms were included in the “limbs” AOI. For dog and cat pictures, the buttocks included the tail, and the forelimbs and hindlimbs were combined into the limbs AOI. For the analysis comparing stimuli with and without a hand-sign, only the gaze at hands were incorporated (Figure 2d). To avoid errors in gaze estimation, the AOI frame was drawn larger than the actual outline (approximately 50 pixels on the edge). From monocular gaze data, the number of fixations and total fixation durations within each AOI per presentation were calculated (Figure 3). For each picture, the AOI to which the first fixation was directed (hereafter, first gazed AOI) were also analyzed. For each AOI, the number of pictures in which the first fixation was directed was counted.

The fixation number and total fixation duration for each AOI were analyzed using a Generalized Linear Mixed Model (GLMM) (lmer, lme4 library, freeware package R, Version 2.14.2; R Development Core Team, 2012). The models were constructed using a Poisson distribution for fixation number data, as these were non-negative count data. A Gaussian distribution was used for analyzing total fixation duration, as these were non-negative continuous data [20]. One data point indicated a fixation number or total fixation duration for each picture for each subject. Stimulus species (human, dog, or cat) and AOI (head, body, buttock, limbs) were set as fixed factors. For the stimulus familiarity model, familiarity (familiar or novel) and AOI (head, body, buttock, limbs) were set as fixed factors. In the hand sign analysis, the hand sign conditions (with or without hand signs) and AOI (head, body, buttock, limbs) were set as fixed factors. Subjects were considered a random factor to control for individual differences. The models with and without the target fixed factor were compared based on the Akaike Information Criterion (AIC [20,21]). Model’s significance was tested using a likelihood ration test. For multiple comparisons of the fixed factors, the models that contained every combination of the fixed factors in the different groups were also compared. The number of pictures categorized into each first gazed AOI was analyzed using a chi-square test; if significant, a standardized residual post-hoc analysis was conducted to investigate any difference from chance frequency.

## 3. Results

### 3.1. Species

**Figure 4:**
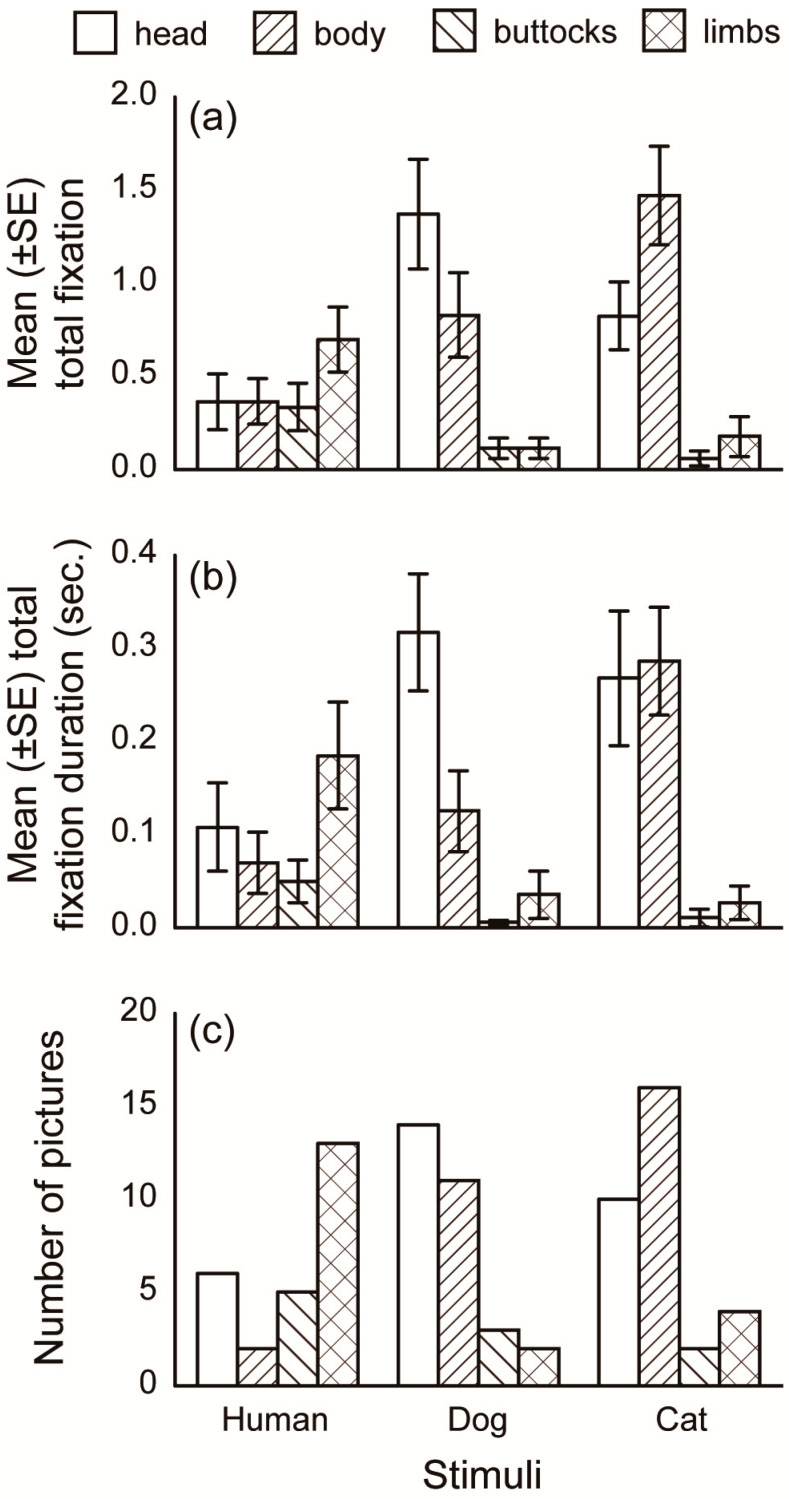
Mean (± SE) fixation number, mean (± SE) total fixation duration, and number of pictures categorized into each first gazed AOI for each picture species.

The most gazed AOI differed between the stimulus species in the fixation number analysis. The fixation number and total fixation duration within each AOI for all three stimulus species are shown in Figure 4a,b, respectively. In the fixation number analysis, the models that included both the stimulus species and AOI showed significantly lower AICs than the models that did not include these factors (Table 2). In the fixation duration analysis, the models that included only the AOI showed significantly lower AICs than the model that include both the stimulus species and AOI. For multiple comparisons of both the fixation number and total fixation duration within the human pictures, the models that included “limb” AOIs at a different group from the other AOIs had the lowest AICs (164.4 and 16.08). For the dog pictures, the models that included a “head” AOI at a different group from the other AOIs showed the lowest AICs (169.5 and 14.85). For cat pictures, the models that included the “head” and “body” AOIs at a different group from the other AOIs showed the lowest AICs (155.5 and 51.11). The number of pictures categorized into each first gazed AOI is shown in Figure 4c. In all stimulus species, the numbers of pictures of each first gazed AOIs were significantly different (human: χ^2^ = 10.00, *p =* 0.019, dog: χ^2^ = 14.00, *p =* 0.003, cat: χ^2^ = 15.00, *p =* 0.002). For the human pictures, the number of pictures where the first gazed AOI was on the “body” was significantly less (*p* = 0.04), and the “limbs” was significantly greater (*p* = 0.003), than chance. For the dog pictures, first gazed AOI for the “head” was significantly greater (*p* = 0.006), and the “limbs” was significantly less (*p* = 0.02), than chance. For the cat pictures, the first gazed AOI for the “body” was significantly greater (*p* = 0.001), and the “buttocks” was significantly less (*p* = 0.014) than chance. These results suggest that the subjects predominantly oriented toward the “limbs” in human pictures, “head” in dog pictures, and “head” and “body” in cat pictures.

### 3.2. Human Familiarity

**Figure 5:**
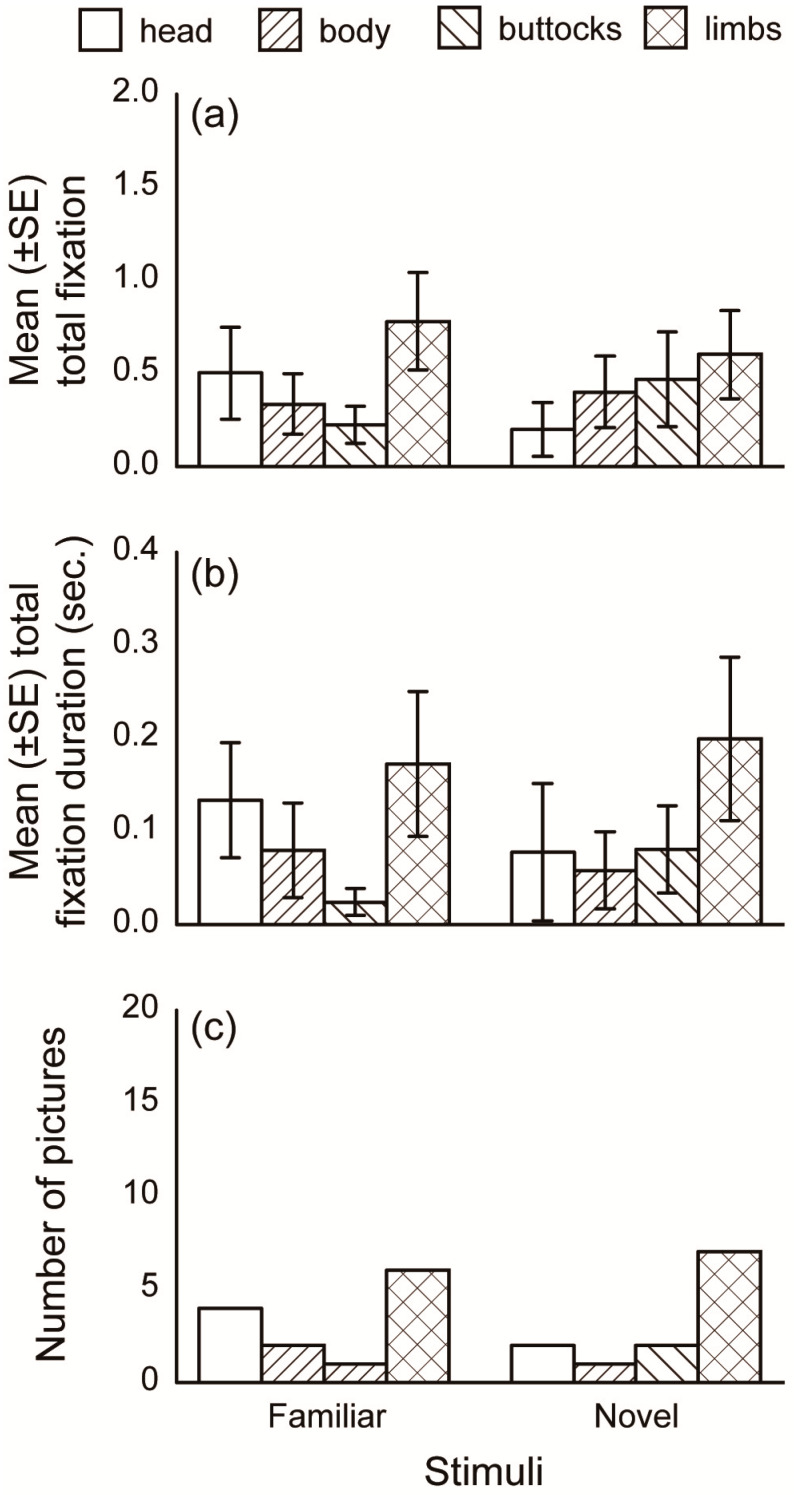
Mean (± SE) fixation number, mean (± SE) total fixation duration, and the number of pictures categorized into each first gazed AOI for the familiar and novel human.

For human pictures, gaze behavior did not differ between familiar and novel individuals. The fixation number and total fixation duration in each AOI for familiar and novel human pictures are shown in Figure 5a,b, respectively. Models that included both familiarity and AOIs did not show a significantly lower AIC than the model that only included the AOIs (Table 2). The number of pictures categorized into each first gazed AOI is shown in Figure 5c. For both the familiar and novel pictures, the number of pictures did not significantly differ between the first gazed AOIs (familiar: χ^2^ = 4.54, *p =* 0.21, novel: χ^2^ = 7.33, *p* = 0.062). These results suggest that familiarity did not affect the dogs’ gaze behavior.

### 3.3. Hand Sign

**Figure 6:**
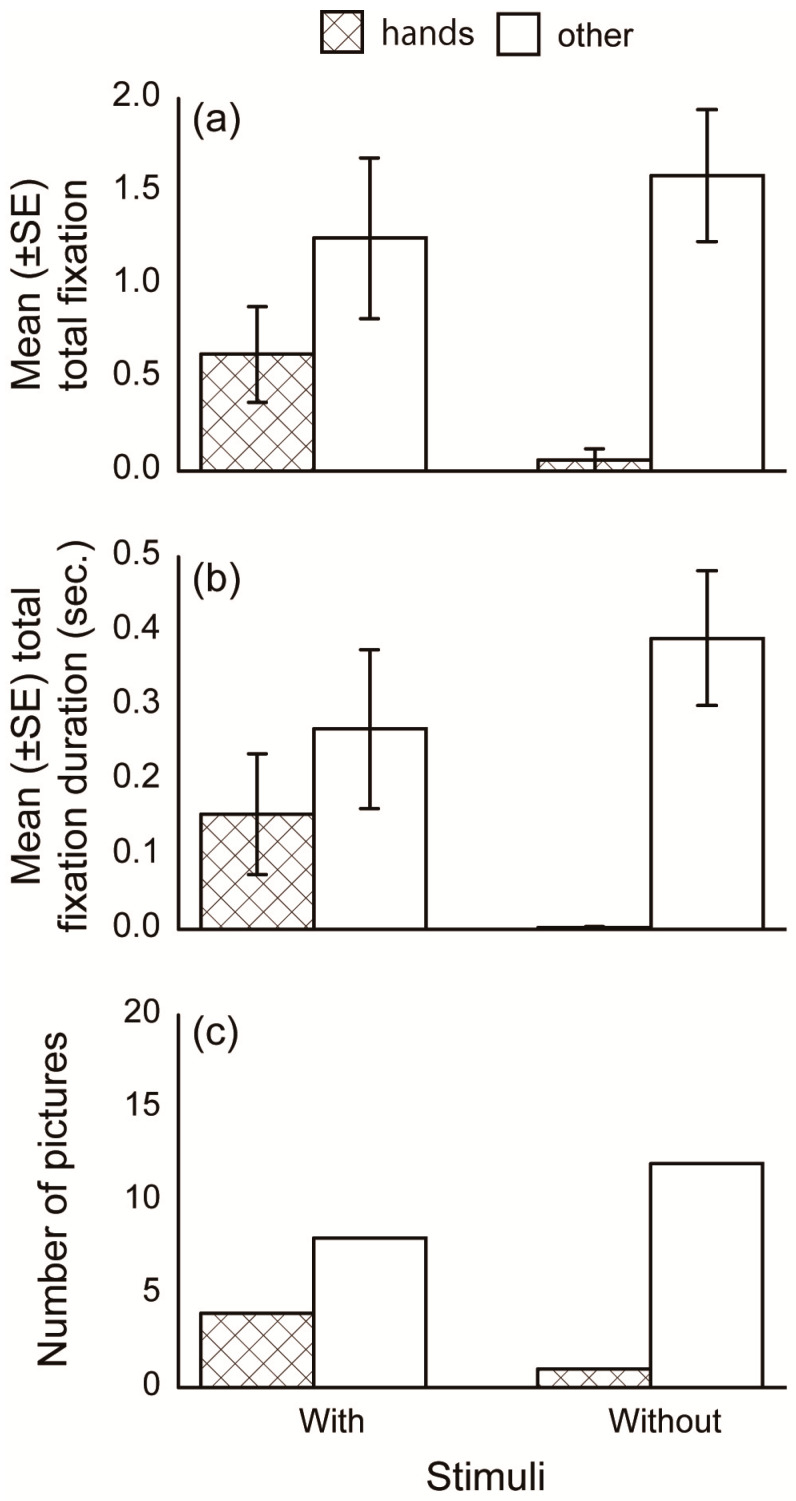
Mean (± SE) fixation number, mean (± SE) total fixation duration, and the number of pictures categorized into each first gazed AOI for human pictures with and without hand signs.

The subject dogs’ gaze behavior differed between the human pictures with and without a hand sign. The fixation number and total fixation duration to hands in the pictures with and without a hand-sign are shown in Figure 6a,b, respectively. For fixation number, the models that included AOIs had significantly lower AICs than models that did not include AOIs (Table 2), although differences between the AICs did not differ significantly in the total fixation duration analysis. The number of pictures in which “hands” and other body parts were gazed first is shown in Figure 6c. For pictures with a hand sign, the number of pictures did not differ significantly between pictures in which “hands” were gazed and other body parts were gazed at first (χ^2^ = 1.33, *p =* 0.248). For pictures without a hand sign, the numbers of pictures of each first gazed AOIs were significantly different (χ^2^ = 9.31, *p* = 0.002). The number of pictures where “hands” were gazed at first was significantly less (*p* = 0.002), and gaze toward other parts of the body was significantly greater (*p* = 0.002), than chance. These results suggest that the dogs gazed at “hand” pictures more so when a hand sign was present.

## 4. Discussion

Dogs’ gaze behavior differed depending on the species presented. The fixation number analyses revealed that the dogs gazed more toward human limbs while also gazing more toward dog heads and cat heads and bodies. The first gazed AOI analysis showed similar results for the human and dog pictures. For the cat pictures, subjects first gazed toward the body. Notable gaze toward human limbs relative to the dog and cat pictures suggests that dogs have acquired an attentional specialization for non-verbal human limb gestures differed with attention pattern when facing with non-human species. Contrary to these analysis, the total fixation duration did not differ among species, suggesting that the subjects directed shorter gaze toward the AOIs with more fixation numbers repeatedly, but longer gaze toward other AOIs. A fixed stare is interpreted as an expression of threat, while gaze alternation is used in cooperative and affiliative interactions [22,23]. The AOIs with more fixation numbers such as human limbs, dog heads, and cat heads might be affiliative signals for dogs. Conversely, when viewing the faces of other dogs, the head and face provide more communicative information. Contrary to our expectations, the subjects gazed toward other dogs’ tails less frequently than the other AOIs. A dog’s tail movement could be modulated by the dog’s emotional state [24]. Although this modulation may be unintentional, dogs are sensitive to other dogs’ tail expressions [25]. However, the present study used still images that lacked information regarding tail movement, which may be why we failed to observe significant attention toward tails in the present study. It should also be noted that our AOIs differed in size, as it was our intent to present pictures depicting natural objects. Interestingly, the subjects gazed more toward the smaller AOIs (e.g., dog head) than the larger AOIs (e.g., dog limbs), indicating that gaze behavior was non-random and contingent on AOI saliency. In other words, the dogs changed their gaze behavior depending on which features were most visually attractive within the particular species presented.

For pictures depicting familiar and novel humans, the fixation number, total fixation duration to each AOI, and first gazed AOIs did not differ significantly. These results indicate that dogs did not change their gaze behavior as a function of stimulus familiarity. There are two potential explanations for this. One possibility is that dogs just do not discriminate between familiar and unfamiliar humans. Alternatively, dogs might discriminate familiarity, but this does not impact their general gaze behavior. Adachi et al. [26] demonstrated that pet dogs have a cross-modal (voice-face) representation of their owners. Somppi et al. [13] also tested whether dogs gaze differently toward personally familiar pictures and found that familiar faces attract attention more than unfamiliar ones. This aforementioned evidence indicates that dogs are able to discriminate novel from familiar human faces. However, in our study, the human stimulus features may have impacted performance. For instance, the images were relatively small, and each human model was wearing the same white lab coat. This may have made it difficult for the dogs to discriminate the novel from the familiar. Hence, differences in gaze patterns on the entirety of a human image within novel and familiar stimuli should be tested in future studies (particularly with a head-mounted eye-tracker; Williams et al. [27] examined gaze patterns toward real people or life-sized images projected on a screen (see [28]).

The fixation number varied significantly as a function of presenting a human hand sign. Here, the dogs gazed more toward human hands when a hand sign was present compared to when it was not. This was also the case for the first gazed AOI. However, total fixation duration did not differ significantly between the hand sign conditions, but a trend level tendency in line with the other gaze analyses was observed. These results suggest that a dog’s attention is enhanced when non-verbal hand signals are present, indicating that hand signs serve as a reliable communication aid for pet dogs. Humans and chimpanzees gaze more at human faces than other parts [6]. Hattori et al. [29] revealed humans and chimpanzees gaze less at humans’ hands conveying human intentional signs. The discrepancy between the results from these study on humans and chimpanzees and those from our study on dogs could be due to a long phylogenetic distance from dogs to human and chimpanzees. During the process of domestication, dogs might have acquired specialized attentional patterns to discern social information from a human hand sign.

This study aimed to clarify dogs’ attentional patterns to the signals produced by human hands by comparing visual gaze toward still pictures showing human, dogs, and cats. The subjects in the experiment were pet dogs, because any potential influence of pre-training to participate in any other task than this experiment must be excluded. This limited the number of the subjects and the breeds of the subject dogs, but the present study could successfully provide some useful insights on the dogs’ domestication process. Following the previous eye-tracking studies in dogs, this study used still pictures as experimental stimuli. This favorably highlighted dogs’ selective attention to human hand signs. However, human bodily signals are usually expressed with movement which was lacking in the still images. As well as information presented in the experimental stimuli, body movement might be important for dogs to communicate with humans. Further experiments with movie stimuli may enable deeper discussions on dogs’ visual cognition specialized to communication with humans.

The present experiment revealed that dogs gazed most notably toward human limbs, and this differed depending on the presence of a hand sign. It appears that dogs have acquired an attentional style that is differed when facing with human and non-human species and enables the collection of information for adequately communicating with people living in their community. Thus, the present study empirically demonstrates that a dog’s cognitive abilities can be adjusted to accommodate life with humans.

## Figures and Tables

**Table 1:** Subject dog list.

ID	Breed	Age (Years)	Sex	Neutering
1 ^a^	Labrador retriever	13	Male	Neutered
2 ^a^	Norfolk terrier	5	Female	Neutered
3 ^a^	Mixed ^c^	2	Male	Neutered
4 ^b^	Unidentified ^d^	18	Female	Neutered
5 ^b^	Shetland sheepdog	9	Male	Neutered
6 ^b^	German shepherd	2	Male	Neutered
7 ^b^	Mixed ^e^	1	Female	Intact

^a^ The dogs who passed the calibration procedure and participated in the experiment. ^b^ The dogs who did not pass the calibration procedure and were omitted from the experiment. ^c^ Cavalier King Charles Spaniel x Mixed ^d^ The breed was not identified because the dogs was a shelter dog and assumed as a golden retriever. ^e^ Chihuahua x Dachshund.

**Table 2:** Generalized Linear Mixed Model results.

	Responsible Factor					
	Fixation number			Total fixation duration		
		Likelihood ratio test			Likelihood ratio test	
Fixed factor	AIC ^a^	χ^2^	*p*	AIC ^a^	χ^2^	*p*
Analysis comparing the three species						
Species and AOI	491.0			106.9		
AOI	547.8	72.80	<0.001	93.67	34.21	<0.001
Species	623.0	149.97	<0.001	121.8	69.25	<0.001
Analysis comparing human familiarity						
Familiarity and AOI	172.2			46.3		
AOI	168.3	4.09	0.39	27.0	1.08	0.90
Familiarity	170.3	10.02	0.12	21.3	7.07	0.31
Analysis comparing stimuli with and without a hand sign						
Hand-sign and AOI	97.1			59.42		
AOI	102.8	9.77	0.008	53.35	3.11	0.21
Hand-sign	126.7	33.6	<0.001	62.72	12.8	0.002

^a^ AIC = “Akaike Information Criterion,” an index used to compare the fitted models. The model with the lower AIC is preferred.

## References

[1] Miklósi Á., Topál J. (2013). What does it take to become ‘best friends’? Evolutionary changes in canine social competence. Trends Cogn. Sci..

[2] Pitteri E., Mongillo P., Carnier P., Marinelli L., Huber L. (2014). Part-based and configural processing of owner’s face in dogs. PLoS ONE.

[3] Miklósi Á. (2007). Dog Behavior, Evolution, and Cognition.

[4] Virány Z., Gácsi M., Kubinyi E., Topál J., Belényi B., Ujfalussy D., Miklósi Á. (2008). Comprehension of human pointing gestures in young human-reared wolves (*Canis lupus*) and dogs (*Canis familiaris*). Anim. Cogn..

[5] Winters S., Dubuc C., Higham J.P. (2015). Perspectives: The looking time experimental paradigm in studies of animal visual perception and cognition. Ethology.

[6] Kano F., Tomonaga M. (2009). How chimpanzees look at pictures: A comparative eye-tracking study. Proc. R. Soc. B-Biol. Sci..

[7] Kano F., Tomonaga M. (2010). Face scanning in chimpanzees and humans: Continuity and discontinuity. Anim. Behav..

[8] Kano F., Call J., Tomonaga M. (2012). Face and eye scanning in gorillas (*Gorilla gorilla*), orangutans (*Pongo abelii*), and humans (*Homo sapiens*): Unique eye-viewing patterns in humans among hominids. J. Comp. Psychol..

[9] Schwarz J.S., Sridharan D., Knudsen E.I. (2013). Magnetic tracking of eye position in freely behaving chickens. Front. Syst. Neurosci..

[10] Tyrrell L.P., Butler S.R., Yorzinski J.L., Fernández-Juricic E. (2014). A novel system for bi-ocular eye-tracking in vertebrates with laterally placed eyes. Methods Ecol. Evol..

[11] Yorzinski J.L., Patricelli G.L., Babcock J.S., Pearson J.M., Platt M.L. (2013). Through their eyes: Selective attention in peahens during courtship. J. Exp. Biol..

[12] Somppi S., Törnqvist H., Hänninen L., Krause C., Vainio O. (2012). Dogs do look at images: Eye tracking in canine cognition research. Anim. Cogn..

[13] Somppi S., Törnqvist H., Hänninen L., Krause C.M., Vainio O. (2014). How dogs scan familiar and inverted faces: An eye movement study. Anim. Cogn..

[14] Somppi S., Törnqvist H., Kujala M.V., Hänninen L., Krause C.M., Vainio O. (2016). Dogs evaluate threatening facial expressions by their biological validity—Evidence from gazing patterns. PLoS ONE.

[15] Kis A., Hernádi A., Miklósi B., Kanizsár O., Topál J. (2017). The way dogs (*Canis familiaris*) look at human emotional faces is modulated by oxytocin. An eye-tracking study. Front Behav. Neurosci..

[16] Téglás E., Gergely A., Kupán K., Miklósi Á., Topál J. (2012). Dogs’ gaze following is tuned to human communicative signals. Curr. Biol..

[17] Törnqvist H., Somppi S., Koskela A., Krause C.M., Vainio O., Kujala M.V. (2015). Comparison of dogs and humans in visual scanning of social interaction. R. Soc. Open Sci..

[18] Pryor K. (1984). Don’t Shoot the Dog!: The New Art of Teaching and Training.

[19] Siniscalchi M., D’Ingeo S., Fornelli S., Quaranta A. (2016). Relationship between visuospatial attention and paw preference in dogs. Sci. Rep..

[20] Dobson A.J. (2002). An Introduction to Generalized Linear Models.

[21] Akaike H. (1974). A new look at the statistical model identification. IEEE Trans. Autom. Control.

[22] Topál J., Miklósi Á., Gácsi M., Dóka A., Pongrácz P., Kubinyi E., Virány Z., Csányi V., Brockmann H.J., Roper T.J., Naguib M., Wynne-Edwards K.E., Mitani J.C., Simmons L.W. (2009). The dog as a model for understanding human social behavior. Advances in the Study of Behavior.

[23] Vas J., Topál J., Gácsi M., Miklósi Á., Csányi V. (2005). A friend or an enemy? Dogs’ reaction to an unfamiliar person showing behavioural cues of threat and friendliness at different times. Appl. Anim. Behav. Sci..

[24] Quaranta A., Siniscalchi M., Vallortigara G. (2007). Asymmetric tail-wagging responses by dogs to different emotive stimuli. Curr. Biol..

[25] Siniscalchi M., Lusito R., Vallortigara G., Quaranta A. (2013). Seeing left- or right-asymmetric tail wagging produces different emotional responses in dogs. Curr. Biol..

[26] Adachi I., Kuwahata H., Fujita K. (2007). Dogs recall their owner’s face upon hearing the owner’s voice. Anim. Cogn..

[27] Williams F.J., Mills D.S., Guo K. (2011). Development of a head-mounted, eye-tracking system for dogs. J. Neurosci. Methods.

[28] Péter A., Miklósi Á., Pongrácz P. (2013). Domestic dogs’ (*Canis familiaris*) understanding of projected video images of a human demonstrator in an object-choice task. Ethology.

[29] Hattori Y., Kano F., Tomonaga M. (2010). Differential sensitivity to conspecific and allospecific cues in chimpanzees and humans: A comparative eye-tracking study. Biol. Lett..

